# Reduced miR-203 predicts metastasis and poor survival in esophageal carcinoma

**DOI:** 10.18632/aging.102543

**Published:** 2019-12-16

**Authors:** Rongqi He, Jintian Wang, Kai Ye, Jiabin Du, Junxing Chen, Weinan Liu

**Affiliations:** 1 Department of Oncology Surgery, Second Affiliated Hospital of Fujian Medical University, Quanzhou, P.R. China; 2 First Hospital of Quanzhou Affiliated to Fujian Medical University, Quanzhou, P.R. China

**Keywords:** esophageal carcinoma, miR-203, KIF5C

## Abstract

We analyzed data from two non-coding RNA profiling arrays made available by the Gene Expression Omnibus (GEO) and found 17 miRNAs with remarkable differential expression between malignant and normal esophageal tissue. Correlation analysis between expression of these 17 miRNAs and patients’ clinicopathological characteristics showed that miR-203 was down-regulated in esophageal carcinoma (EC) tissues and was significantly associated with lymph node metastasis and poor overall survival. Overexpression of miR-203 significantly attenuated cellular proliferation, migration and invasion by EC cells in culture. Additionally, gene expression profiles and bioinformatics analysis revealed KIF5C to be a direct target of miR-203, and KIF5C overexpression partially counteracted the tumor inhibitory effects of miR-203 on EC cells. We also observed that miR-203, reduced KIFC5 protein levels, promoted cytoplasmic accumulation of Axin2, and reversed the invasive phenotype of EC cells. Taken together, these data demonstrate that miR-203 is a tumor suppressor in EC cells and its expression level could potentially be used as a prognostic indicator for EC patient outcomes.

## INTRODUCTION

EC is one of the most common cancers worldwide [1, 2]; due to a high incidence of recurrence, its prognosis is poor [3]. miRNAs play a critical role in regulating tumorigenesis and metastasis of EC [4, 5], and some miRNAs have been identified as prognostic markers or potential therapeutic targets [6, 7]. miRNAs can act as either oncogenes or tumor suppressors to modulate growth, angiogenesis, drug or chemo-resistance, invasion, and metastasis of malignant cells [8, 9]. Different cancers exhibit specific miRNA expression signatures (miRNome), which characterize the malignant state and define some of their clinicopathological features [10]. Thus, the distorted and unique expression profiles of miRNAs make them sensitive biomarkers for clinical diagnosis and prognosis of cancers.

The complexity of miRNA-mRNA interactions and the emergence of competing endogenous RNAs (ceRNAs) [11, 12] complicate our understanding of the role and clinical value of miRNAs in EC. A systematic non-coding RNA profiling array and miRNA-seq evaluation in EC development may implicate certain miRNAs as prognostic markers and reveal potential therapeutic targets.

In this study, we used non-coding RNA profiling array data released by GEO [13] to identify miRNAs differentially expressed between malignant and normal esophageal tissue. We performed correlation analysis with clinicopathological and miRNA-sequencing data of tumor tissues released by the Cancer Genome Atlas (TCGA) [14] to find miRNAs with significantly altered expression in esophageal carcinoma tissues and evaluated correlations to patient outcomes in order to find predictors for EC prognostic evaluation.

## RESULTS

### miR-203 predicts overall survival of EC patients

**Figure 1:**
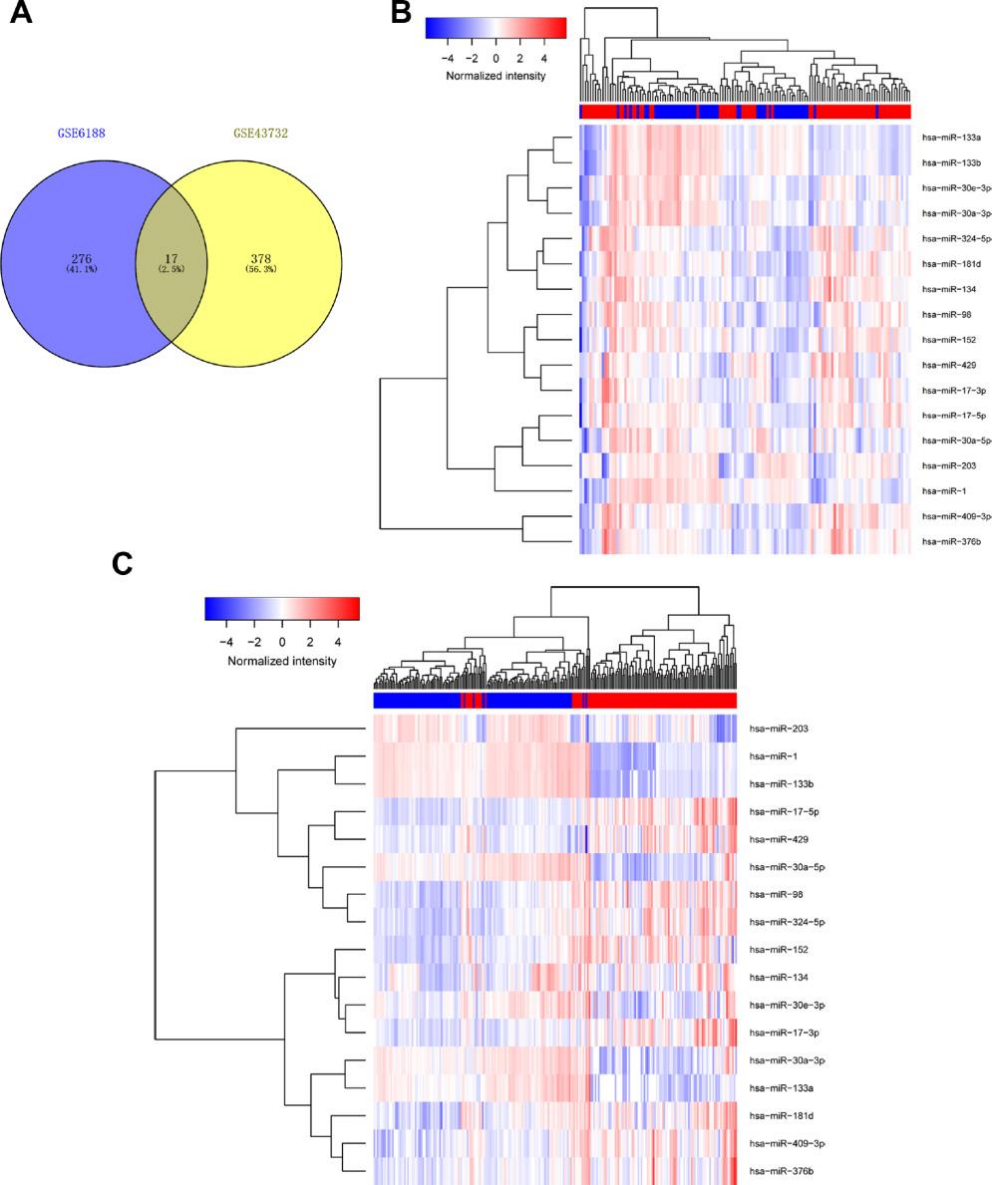
**Determination of differentially expressed miRNAs.** We analyzed data of two non-coding RNA profiling arrays (ID: GSE43732, GSE6188). Differentially expressed miRNAs with large changes (p< 0.05, fold Chang (FC)>1.5) in both arrays were considered to be significantly altered. Results are shown in Venn diagram (**A**) and heat map (**B**).

To identify miRNAs with prognostic potential in EC, we first analyzed data from two non-coding RNA profiling arrays released by GEO (ID: GSE43732, GSE6188) [15, 16]. Briefly, one comparison (cancer tissue vs adjacent normal tissue) was conducted in each of two arrays to find miRNAs which underwent expression changes from normal to malignant tissue. Differentially expressed miRNAs with larger changes (P< 0.05, Fold Change (FC)＞1.5) [17] in both arrays were considered to be significantly altered. We found 17 miRNAs with remarkable differential expression between malignant and normal esophageal tissue, including miR-98, miR-133, and miR-30a-3 (Figure 1, Table 1 and Supplementary Tables 1, 2).

**Table 1:** Remarkable differential expression of miRNAs between malignant esophageal tissue and normal.

**ID**
hsa-miR-1
hsa-miR-133a
hsa-miR-133b
hsa-miR-134
hsa-miR-152
hsa-miR-17-3p
hsa-miR-17-5p
hsa-miR-181d
hsa-miR-203
hsa-miR-30a-3p
hsa-miR-30a-5p
hsa-miR-30e-3p
hsa-miR-324-5p
hsa-miR-376b
hsa-miR-409-3p
hsa-miR-429
hsa-miR-98

**Figure 2:**
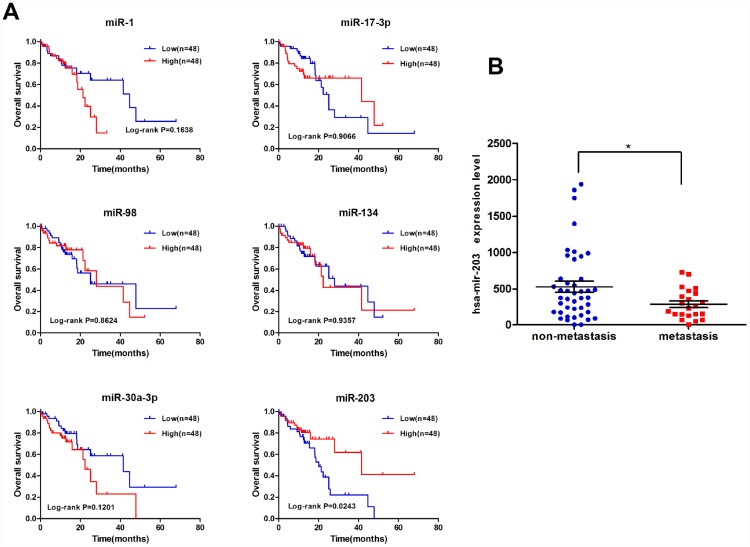
**miR-203 is correlated with overall survival of EC patients.** (**A**) Kaplan-Meier curves for overall survival according to differential expression level of miRNAs in EC patients; cutoff value is the average expression level. p-value was calculated based on log rank test. (**B**) The significant association between lymph node metastasis and miRNA-203 expression level.

We performed correlation analysis between expression of these 17 miRNAs and clinicopathological characteristics on miRNA-sequencing data of tumor tissues of 97 EC patients from TCGA. As shown in Figure 2 and Supplementary Figure 1, only the expression of miR-203 was correlated with overall survival of EC patients. miR-203 was also significantly associated with lymph node metastasis (Figure 2B); we thus focused our efforts to evaluate the role of miR-203 in EC.

### miR-203 suppresses migration and invasion of EC cell lines

**Figure 3:**
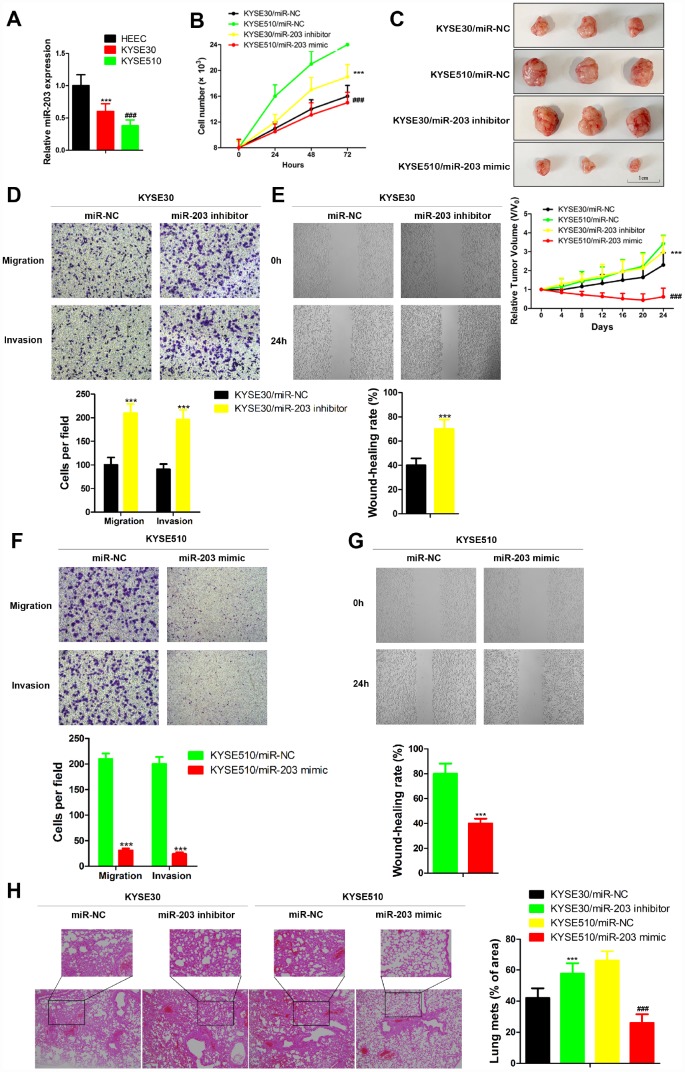
**miR-203 inhibits proliferation, migration and invasion of EC cells *in vivo* and *in vitro*.** (**A**) miR-203 expression in normal esophageal cell line HEEC and EC cell lines KYSE30 and KYSE510 was determined by qRT-PCR. Data is presented as mean ± SD from three independent experiments. ***p < 0.001 (HEEC vs. KYSE30), ^###^p < 0.0001 (KYSE510 vs. KYSE30). (**B**) Numbers of KYSE30 cells and KYSE510 cells were counted at indicated time post transfected with miR-203 inhibitor, miR-203 mimic or miR-203 negative control (miR-NC). Data is presented as mean ± SD from three independent experiments. ***p<0.001 (KYSE30/miR-203 inhibitor vs. KYSE30/miR-NC), ^###^p<0.0001 (KYSE510/miR-203 mimic vs. KYSE510/miR-NC). (**C**) Tumor volume changes of mice with different genetically modified cells. Data are presented as mean ± SD (n=10). ***p<0.001 (KYSE30/miR-203 inhibitor vs. KYSE30/miR-NC), ^###^p<0.0001 (KYSE510/miR-203 mimic vs. KYSE510/miR-NC). Cellular migration and invasion of KYSE30 cells were evaluated by transwell assays (**D**) and wound healing assay (**E**). Cellular migration and invasion of KYSE510 cells were evaluated by transwell assays (**F**) and wound healing assay (**G**). Quantification of the numbers of migrating or invading cells is presented as mean ± SD from three independent experiments (×100). ***p<0.01 (vs. negative control miR-NC). (**H**) The changes of lung metastasis of different genetically modified cells. Data is presented as mean ± SD from three independent experiments (×100). ***p<0.001 (KYSE30/miR-203 inhibitor vs. KYSE30/miR-NC), ^###^p<0.0001 (KYSE510/miR-203 mimic vs. KYSE510/miR-NC).

Here, we analyzed the expression of miR-203 in two human EC cell lines with different metastatic potential, KYSE30 (less metastatic potential) and KYSE510 (greater metastatic potential) [18]. The level of miR-203 was significantly decreased in both KYSE30 and KYSE510 compared to the normal esophageal cell line, HEEC (Figure 3A). Furthermore, KYSE510 cells had a much lower level of miR-203 than KYSE30 cells (Figure 3A). Then, KYSE30 and KYSE510 cells were transfected with miR-203 mimic or inhibitor. miR-203 inhibitor significantly increased proliferation of EC cells and introduction of miR-203 mimic led to an obvious defect in cell proliferation compared to the miR-NC group both *in vivo* and *in vitro* (Figure 3B and 3C). *in vitro* transwell and wound healing assays as well as *in vivo* experimental pulmonary metastasis assays showed that miR-203 inhibitor vigorously enhanced migration, invasion and pulmonary metastasis of KYSE30 cells, while miR-203 mimic produced the opposite results in KYSE 510 cells (Figure 3D–3H). In addition, miR-203 mimic almost completely inhibited *in vivo* experimental pulmonary metastasis of KYSE30 cells, while treatment with miR-203 inhibitor reduced pulmonary metastasis of KYSE510 cells (Supplementary Figure 3).

### KIF5C is a direct target of miR-203

**Figure 4:**
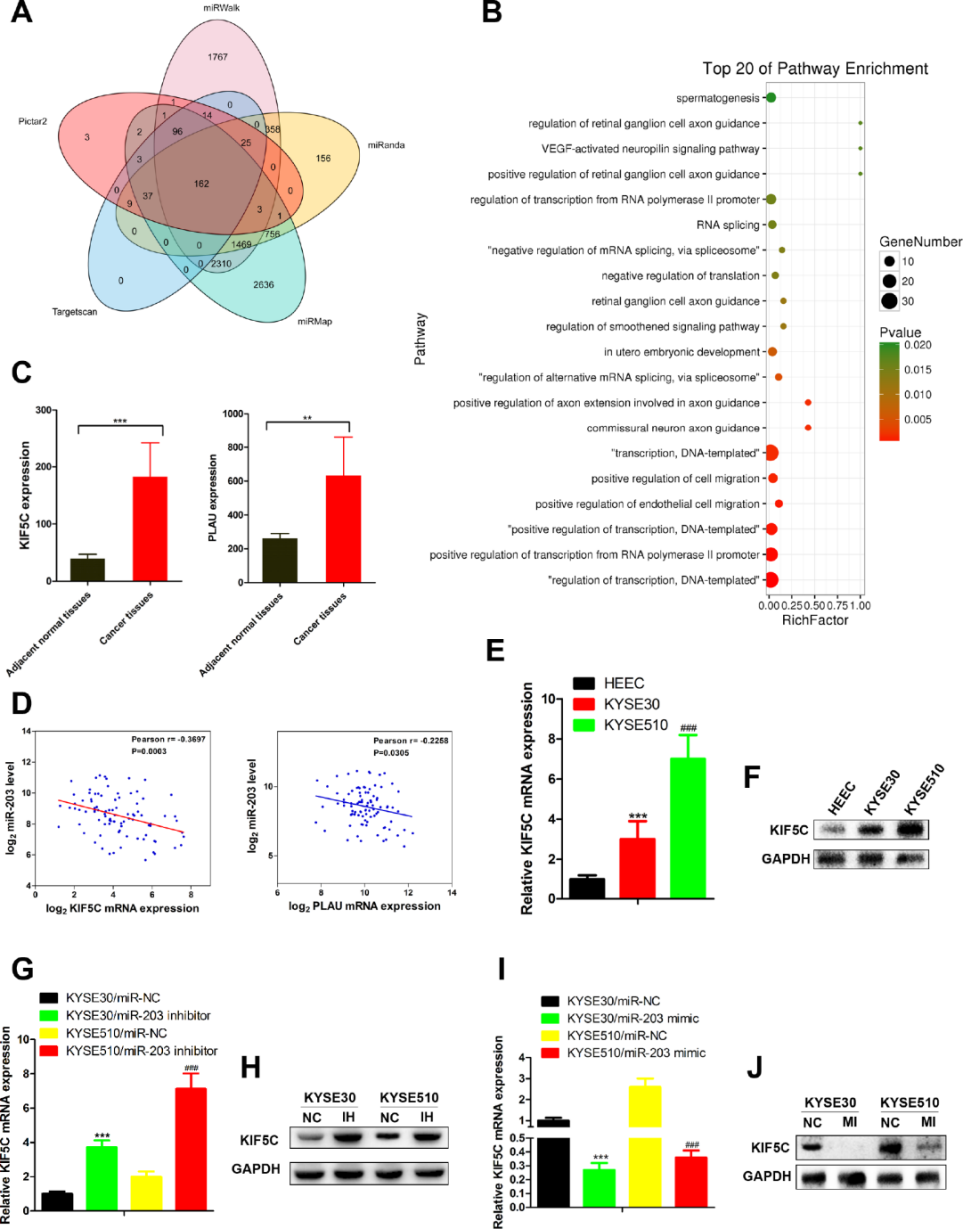
**miR-203 inhibits migration and invasion of EC cells through directly targeting KIF5C expression.** (**A**) The targets of miR-203 in EC predicted by TargetScan7.1. (**B**) Pathway enrichment of 162 potential targets of miR-203. (**C**) mRNA expression of potential direct targets in tumor tissues and adjacent normal tissues of EC patients was revealed by mRNA-seq provided by TCGA. Data is presented as mean ± SD. ***p < 0.001, **p < 0.01 (vs. adjacent normal tissues). (**D**) Scatterplot depicts a significant inverse correlation between miR-203 and KIF5C mRNA expression. KIF5C expression in normal esophageal cell line HEEC and EC cell lines KYSE30 and KYSE510 was determined by qRT-PCR (**E**) and western blotting (**F**). GAPDH was used as an internal control. Data is presented as mean ± SD from three independent experiments. ***p < 0.001 (HEEC vs. KYSE30), ^###^p < 0.0001 (KYSE510 vs. KYSE30). Effect of miR-203 inhibitor on KIF5C mRNA (**G**) and protein (**H**) expression was determined in EC cells. Effect of miR-203 mimics on KIF5C mRNA (**I**) and protein (**J**) expression was determined in EC cells. Data is displayed as the Mean ± SD of three independent experiments. ***p < 0.001, ^###^p < 0.001. (vs. miR-NC: miR-203 negative control).

To identify which miR-203 targets were responsible for its effects on cancer cell migration and invasion, we predicted the function of miR-203 by TargetScan7.1 with very high stringency enrichment analysis [19] and found 162 genes predicted as potential targets of miR-203 (Figure 4A). Among the 162 potential targets, only SEMA6D, VEGFA, KIF5C, CBLL1, FGF1, PIK3R1, PLAU, CCAR1 were connected with positive regulation of cell migration by gene ontology (GO) pathway enrichment analysis (Figure 4B and Supplementary Table 3). We then analyzed the mRNA expression of these 8 genes in RNA-seq data of EC patients from TCGA data portal. Interestingly, only PLAU and KIF5C were elevated in cancer tissues compared to adjacent normal tissues (Figure 4C and Supplementary Figure 2). Correlation analysis showed that KIF5C inversely correlated significantly (p＜0.01) with miR-203 expression in EC patients, while PLAU did not (Figure 4D).

Next, we measured KIF5C mRNA and protein expression in EC cell lines in response to miR-203 mimic or inhibitor treatment. Contrary to miR-203 expression, both mRNA and protein expression of KIF5C were increased in EC cells compared with normal cells (Figure 4E, 4F). miR- 22 inhibitor vigorously up-regulated KIF5C in KYSE30 and KYSE510 cells (Figure 4G, 4H). In response to miR-203 mimic treatment, we observed a significant down-regulation of KIF5C mRNA and protein levels in both KYSE30 and KYSE510 cells (Figure 4I, 4J). Overall, these results demonstrate that KIF5C is a direct target of miR-203 in EC cells.

### miR-203 reduces cell migration and invasion by targeting KIF5C

**Figure 5:**
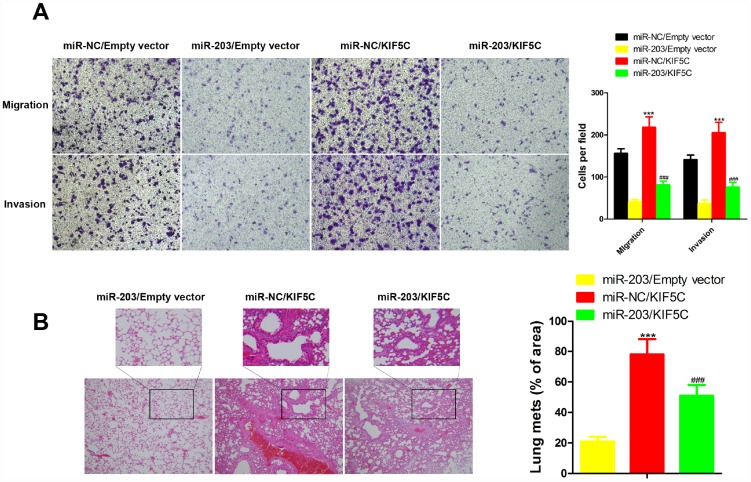
**Overexpression of KIF5C partially rescued the inhibitory effects of miR-203 on migration and invasion of EC cells *in vitro* and *in vivo*.** *in vitro* transwell assay (**A**) and *in vivo* experimental pulmonary metastasis assay (**B**). Data is displayed as the Mean ± SD. ***p < 0.001 (miR-NC/KIF5C vs. miR-NC/Empty vector), ^###^p < 0.001 (miR-203/KIF5C vs. miR-203/Empty vector).

To clarify the importance of KIF5C for miR-203-mediated inhibition of cell migration and invasion. KYSE510 cells were transfected with KIF5C-expressing plasmid with or without miR-203 mimic. We observed that the inhibitory effects of miR-203 mimic on cell migration and invasion of KYSE510 cells were partially compensated by overexpression of KIF5C in transwell and wound healing assay (Figure 5A). Our *in vivo* assay showed the same results (Figure 5B). These data suggest that KIF5C down-regulation might be one important cause for the decrease in cell migration and invasion observed upon miR-203 overexpression.

### miR-203 inhibits β-catenin signaling

**Figure 6:**
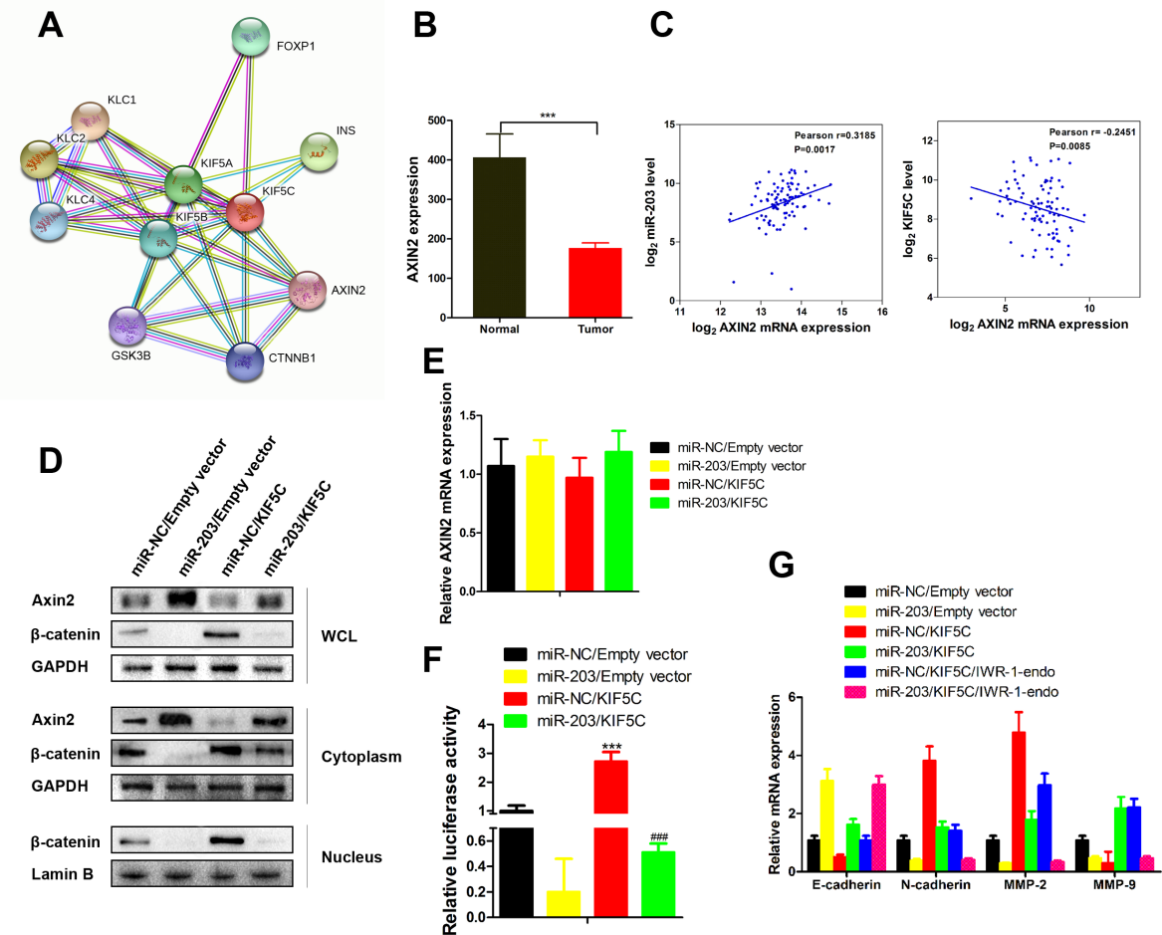
**miR-203 promotes nuclear expression of β-catenin via enhancing Axin2 expression.** (**A**) Proteins interacted with AXIN2 and KIF5C was predicted by String database (http://string-db.org). (**B**) AXIN2 mRNA expression level in tumor tissues and adjacent normal tissues of EC patients. Data is presented as mean ± SD. ***p < 0.001 (vs. adjacent normal tissues). (**C**) Scatterplot depicts a significant inverse and positive correlation between AXIN2 and KIF5C, miR-203 mRNA expression, respectively. (**D**) KYSE510 cells were transfected with miR-203 mimic or KIF5C recombinant plasmid. 48 h later, protein expression of Axin2, β-catenin in different cellular components were detected by western blotting. (**E**) AXIN2 mRNA expression in response to miR-203 mimic and KIF5C overexpression was determined by qRT-PCR. (**F**) Transcriptional activity of β-catenin has been determined by luciferase reporter gene assay. Data is presented as mean ± SD. ***p < 0.001 (miR-NC/KIF5C vs. miR-NC/Empty vector), ^###^p < 0.001 (miR-203/KIF5C vs. miR-203/Empty vector). (**G**) In some cases, KYSE510 cells were pretreated with IWR-1-endo (β-catenin pathway inhibitor) for 1 h, and then transfected with miR-203 mimic or KIF5C-expressing plasmid for 48 h. mRNA expressions of E-caherin, N-cadherin, MMP2 and MMP9 were detected by qRT-PCR. Datas are displayed as the Mean ± SD of three independent experiments.

As an important tumor suppressor, the level of Axin2 can be increased by KIF5C (Figure 6A). In EC patient samples the level of Axin2 was decreased (Figure 6B). Correlation analysis further showed that Axin2 associated positively with miR-203 expression and negatively with KIF5C expression (Figure 6C). We next examined the effects of miR-203 and KIF5C on Axin2 expression in KYSE510 cells. Overexpression of KIF5C caused decreased total and cytoplasmic expression of Axin2 (Figure 6D). However, the effect of KIF5C on Axin2 was markedly reversed in the presence of miR-203 mimic (Figure 6D). Furthermore, it is noteworthy that miR-203 alone did not decease AXIN2 mRNA expression (Figure 6E) in KYSE510 cells. As Axin2 is a known β-catenin inhibitor [20], we further found that miR-203 suppressed KIF5C-induced β-catenin expression and related transcriptional activity (Figure 6D, 6F). Hence, these results suggest that miR-203 inhibits β-catenin activity through promoting cytoplasmic accumulation of Axin2 by suppressing KIF5C.

Overexpression of KIF5C in KYSE510 cells significantly increased of N-cadherin, MMP2 and MMP9 mRNA expression and suppressed E-cadherin expression (Figure 6G). However, the effect of KIF5C on these proteins was markedly reversed in the presence of miR-203 or IWR-1-endo, which induces the level of Axin2 and β-catenin degradation [21]. And, more importantly, miR-203 and IWR-1-endo showed synergistic effects on expression of E-cadherin, N-cadherin, MMP2 and MMP9 (Figure 6G). Thus, our data demonstrate that Axin2 is an essential indirect downstream molecule of miR-203.

## DISCUSSION

In previous studies, multiple dysregulated miRNAs in EC tissues have shown potential value in prognosis and cancer therapy, including miR-125b-5p [22], miR-126 [23] and miR-26 [24]. However, with the raise of miRNA signature and the emerging of ceRNAs [25], the certain role and clinical value of miRNAs in EC remains an ongoing process. In this study, we first analyzed data from two non-coding RNA profiling arrays released by GEO to reveal 17 miRNAs with significant differential expression between malignant and normal esophageal tissue, including miR-98, miR-133, and miR-30a-3. Then, we systematically analyzed miR-seq data of EC patients and confirmed that miR-203 is a potential prognostic predictor for EC outcomes. miR-203 suppressed migration and invasion of EC cells by directly targeting KIF5C for degradation.

miR-203 exhibits a complicated but important role in the development of numerous cancers [26–32]. miR-203 inhibits cellular progression (proliferation, metastasis, tumor angiogenesis) and modulates the tumor microenvironment through targeting multiple oncogenic proteins, such as the p53 pathway; it also exhibits potential as a therapeutic target [29, 33, 34].

Through gene expression profiles, bioinformatics, and correlation analysis, we demonstrated that KIF5C may be the direct target of miR-203 in EC cells. We observed increased KIF5 in EC versus normal esophageal cells and that overexpression of KIF5C significantly compensated for the inhibitory effects of miR-203 on cell migration and invasion of KYSE510 cells both *in vitro* and *in vivo*. We conclude that KIF5C is an important target molecule through which miR-203 suppresses metastasis of EC cells.

The level of Axin2, an important tumor suppressor, can be increased by inhibiting KIF5C (see Figure 6A). We used a series of molecular biological means to demonstrate that miR-203, by suppressing KIF5C, promoted cytoplasmic accumulation of Axin2, thereby inhibiting the protein level and transcriptional activity of β-catenin. Accordingly, we also found that increased miR-203 reduced the expression level of multiple proteins downstream of β-catenin. The exact roles and mechanisms of KIF5C and miR-203 in accumulation of Axin2 need to be clarified in future studies.

**Figure 7:**
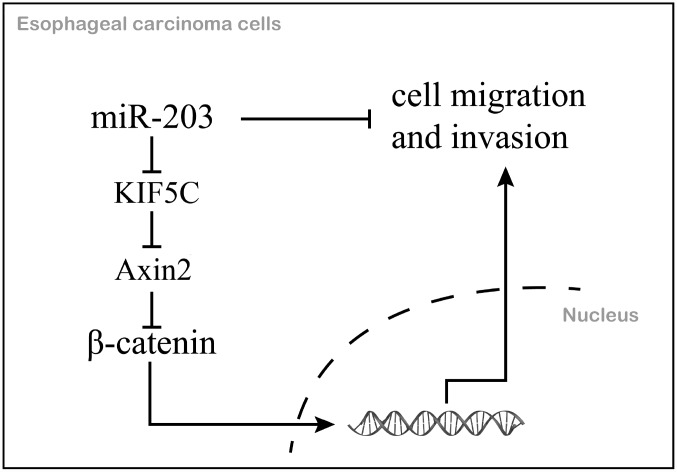
**The mechanism schematic diagram of this study.**

In summary, we identified miR-203 as a prognostic marker for EC. We further demonstrated that miR-203 inhibits cell migration and invasion via directly inhibiting KIF5C expression and thus enhances the antitumor activities of the downstream protein, Axin2 in EC (Figure 7). The features of this miR-203/KIF5C/Axin2 signaling arm support its exploration as a potential therapeutic target or prognostic biomarker for EC.

## MATERIALS AND METHODS

### GEO data analysis

Non-coding RNA profiling array data were downloaded from the GEO (ID: GSE43732, GSE6188). In brief, one comparison (cancer tissue vs. adjacent normal tissue) was conducted in each of two arrays. Differentially expressed miRNAs with significant changes (p< 0.05, fold change (FC)>1.5) in both arrays were considered to be significantly altered.

### TCGA data analysis

Level 3 miRNA expression data (processed/mapped) for EC specimens profiled using Illumina HiSeq along with metafiles annotating each dataset were retrieved from both the miRNA quantification and isoform files available at the TCGA data portal. Level 3 normalized mRNA expression data for EC specimens were obtained using Illumina HiSeq 2000 sequencers by the University of Texas MD Anderson Cancer Center RPPA Core Lab. Coded patient survival data were extracted from the TCGA clinical information file. Permission to access all data was obtained from the Data Access Committee for the National Center for Biotechnology Information Genotypes and Phenotypes Database (dbGAP) at the National Institute of Health. The expression of miRNA and mRNA was normalized and presented as Log2 value. Analysis of all data was done using GraphPad Prism 6 (San Diego, CA, USA).

### RNA isolation and quantitative real-time PCR

Total RNA was extracted from the tissue samples and cells using TRIzol reagent (Invitrogen). First-strand cDNA was synthesized with 500 ng total RNA using a Hiscript® II QRTSuperMix (Vazyme). Quantitative RT-PCR was performed using a SYBR Green Master kit (Bio-Rad. USA) according to the manufacturer’s instructions. The comparative cycle threshold (Ct) method was applied to quantify the expression levels through calculating the 2(-ΔΔCt) method. The primers used for PCR were as follows: GAPDH: 5′- TGTGGGCATCAATGGATTTGG-3′ (forward) and 5′- ACACCATGTATTCCGGGTCAAT-3′ (reverse); KIF5C: 5′- ATCCCACGAATTGCCCATGAT-3′ (forward) and 5′- CCCTTTACATACGGGACTCTGT-3′ (reverse); AXIN2: 5′- TACACTCCTTATTGGGCGATCA-3′ (forward) and 5′- TTGGCTACTCGTAAAGTTTTGGT-3′ (reverse).

### Western blot analysis

Whole-cell lysates were prepared with RIPA buffer containing protease and phosphatase inhibitors. Nuclear and cytoplasmic cell extracts were prepared using the NE-PER Nuclear and Cytoplasmic Extraction kit (Thermo). Equal amounts of cell lysates (50 μg) were loaded on SDS-PAGE and transferred onto PVDF membranes. After membranes were blocked, they were incubated with antibody against GAPDH (1:5000, bioworld), Axin2 (1: 2000, Abcam), KIF5C (1μg/ml, GeneTex), β-catenin (1: 5000, Abcam), MMP-2 (1: 3000, Abcam), MMP-9 (1:3000, Abclonal), E-cadherin (1:500, Abcam) and N-cadherin (1:1000, Abcam) followed by incubation with Goat anti-Rabbit IgG -HRP (1: 10000, Bioworld Biotechnology). Target proteins were detected by the ECL system (Millipore) and visualized with the ChemiDoc XRS system (Bio-Rad).

### Cell culture and transfection

Normal esophageal cell line HEEC and EC cell lines KYSE30 and KYSE510 were maintained in our laboratory and cultured in RPMI1640 (Gibco) containing 10% FBS (Gibco) and 100 units/mL penicillin-streptomycin (Beyotime, Shanghai, China). The cells were seeded in six-well plates and transfected with miR-203 mimics/inhibitor or KIF5C shRNAs or negative control using lipofectamine 3000 transfection reagents (Invitrogen, Carlsbad, CA, USA) following the manufacture’s protocol.

### *in vitro* cell migration and invasion assay [35]

Transwell assay was performed to measure the migration and invasion abilities of transfected KYSE30 and KYSE510 cells. For cell invasion assay, Matrigel (BD Biosciences) was pre-coated to the upper side of the membrane, incubated at 37°C for 1h for gel formation, and hydrated in FBS for two hours before use. The cells were digested and seeded in the upper chamber at a density of 3×10^5^ cells/mL. The lower chamber was filled with medium containing 20% FBS, and then the set was assembled and incubated at 37°C for 36 hours. After incubation, the membrane was stained using 0.1% crystal violet for 30 minutes. Stained cells were washed in PBS, and counted under an optical microscope. For cell migration assay, the same procedures were conducted but without Matrigel on the membrane.

### Wound healing mobility assay [36]

5×10^5^ cells were seeded into a 6-well plate and allowed to grow to confluent monolayer in complete medium. The monolayers were disrupted (i.e., wounded) by scraping them with a P200 micropipette tip, and cellular debris was dislodged by washing with PBS for 3 times. At the indicated time (0 and 24h) after scraping, photographs of the exact wound areas were taken. Each dish was counted three times and the counts were averaged.

### Experimental pulmonary metastasis model [37]

EC cells (2×10^7^ cells/ml in 0.2 ml PBS) were injected into BALB/c nude mice via the lateral tail vein. Following injection, mice were randomly divided into different groups. Animals were weighed every 3 days. All mice were sacrificed at 18 days following tumor injection. Lungs were removed and fixed. Metastatic foci on the surface of lung were photographed.

### Transplanted tumor model [17]

A total of 40 (male BALB/c nude mice aged four- to five-week old and weighing 18–22g were purchased from Vital River Laboratory Animal Technology Co., Ltd. (Beijing, China). The animals were maintained in a pathogen-free facility (23°C ± 2°C, 55%±5% humidity, 12 h light/12 h dark cycle), and then injected subcutaneously with cancer cells (2×10^6^ per mouse) into the abdomen. We then waited 2 weeks to establish the transplanted tumor mouse model. The xenografted mice were randomly divided into four groups, each consisting of 10 mice. The tumor size was measured by Vernier caliper every 4 days, and was calculated by tumor volume = 0.5 × L×W×H, wherein L is the tumor dimension at the longest point, W is the tumor dimension at the widest point, and H is the tumor dimension at the highest point. Relative tumor volumes were calculated as V_t_/V_0_ (V_0_ is the tumor volume when the treatment was initiated).

### Statistical analysis

All experiments were repeated at least three times. Data are presented as mean ± SD and analyzed for significance using GraphPad Prism 6 software (San Diego, CA, USA). Difference between two-groups was assessed using student’s t-test. One-way ANOVA followed by Newman-Keuls post hoc testing (95% confidence) was used to determine difference among more than two groups. The survival analysis was illustrated by Kaplan-Meier curves with log-rank test. p-value of < 0.05 is considered statistically significant.

## Supplementary Material

Supplementary Figures

Supplementary Tables

Supplementary Table 1

Supplementary Table 2

Supplementary Table 3
