# Supplementary material for: Reduced miR-203 predicts metastasis and poor survival in esophageal carcinoma
Source: Aging (Albany NY). 2019 Dec 16;11(24):12114–30. doi: 10.18632/aging.102543 (PMC6949080; doi:10.18632/aging.102543)
Supplement: Supplementary Figures [file aging-11-102543-s004..pdf]

SUPPLEMENTARY FIGURES

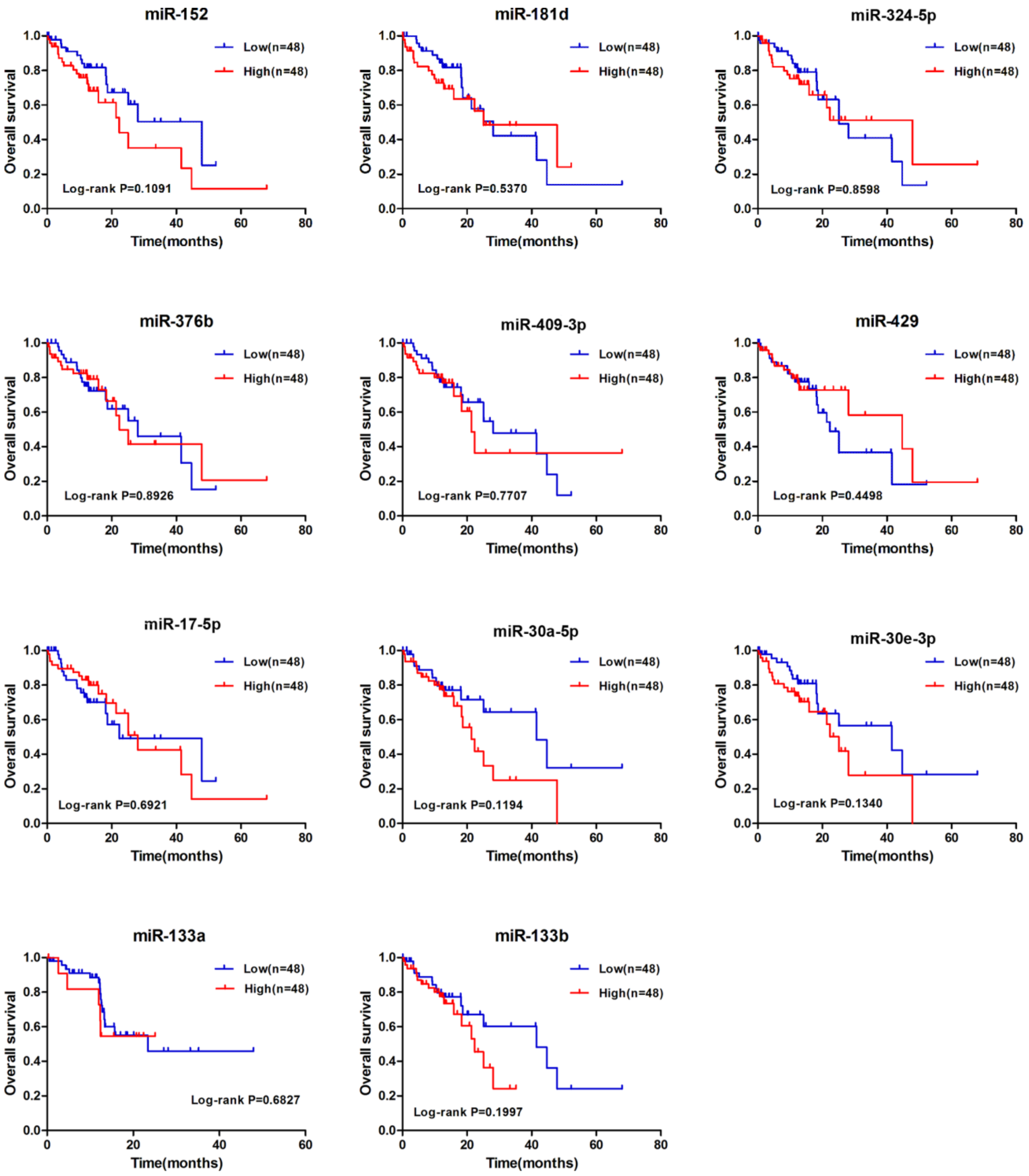

Supplementary Figure 1. Relationship between overall survival and miRNA expression of EC patients. Kaplan-Meier curves for overall survival according to differential expression level of miRNAs in EC patients, cutoff value is the average expression level. p-value was calculated based on log rank test.

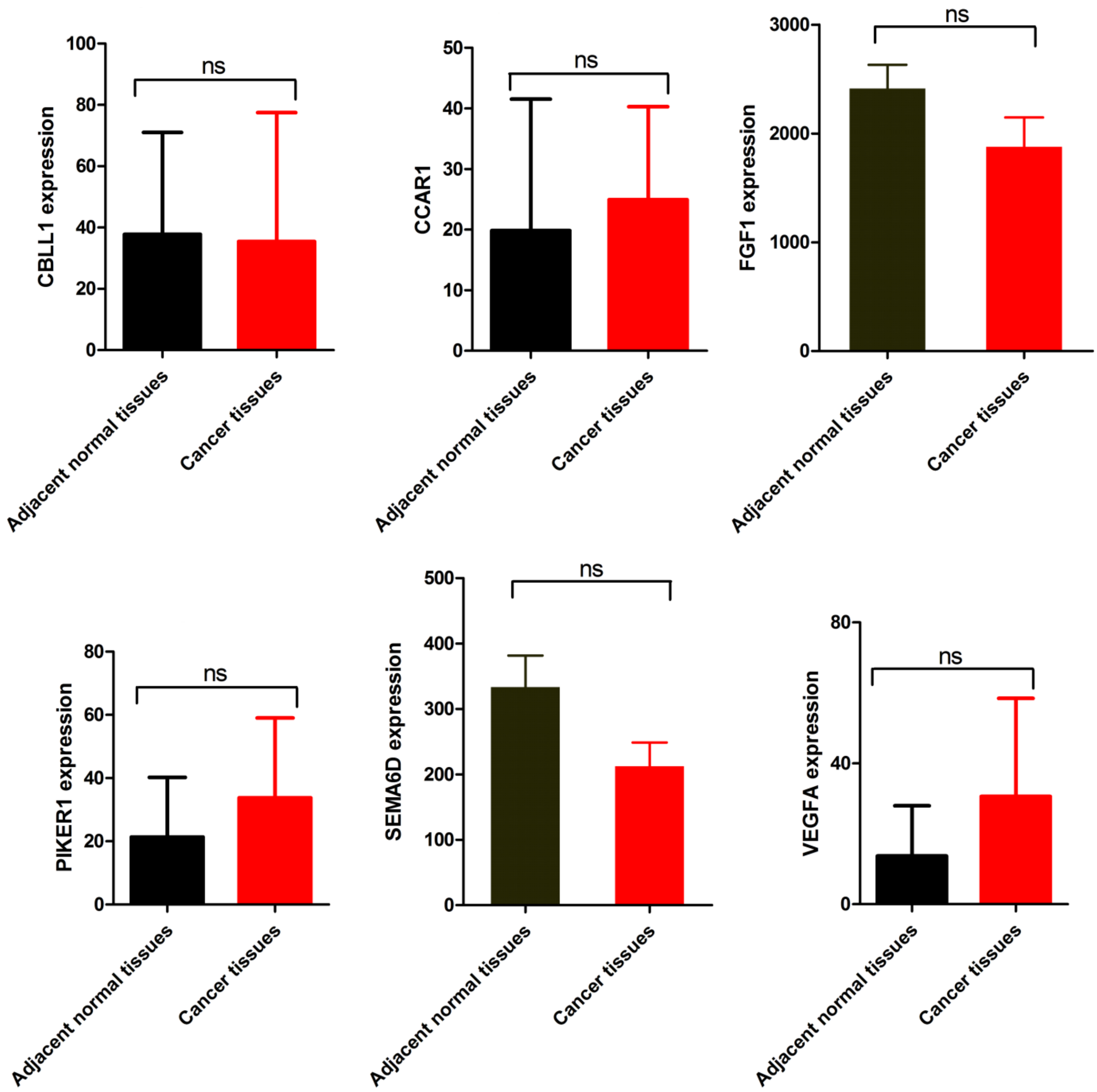

Supplementary Figure 2. mRNA expression of potential direct targets of miR-203 in tumor tissues and adjacent normal tissues of EC patients was revealed by mRNA-seq provided by TCGA. Data are presented as mean ± SD.

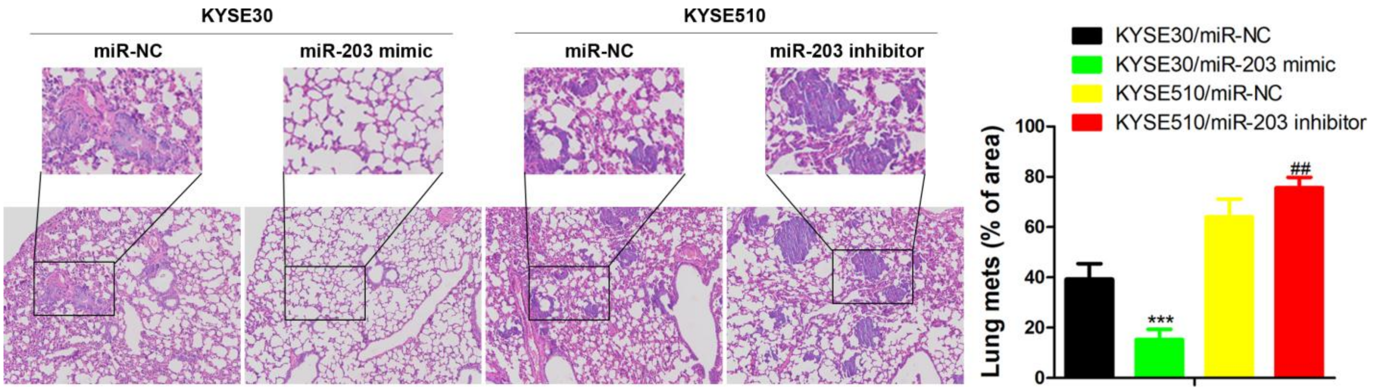

Supplementary Figure 3. The changes of lung metastasis of different genetically modified cells. Data is presented as mean ± SD from three independent experiments (×100). ***p<0.001 (KYSE30/miR-203 mimic vs. KYSE30/miR-NC), #p<0.05 (KYSE510/miR-203 inhibitor vs. KYSE510/miR-NC).
