# Supplementary material for: Reduced miR-203 predicts metastasis and poor survival in esophageal carcinoma
Source: Aging (Albany NY). 2019 Dec 16;11(24):12114–30. doi: 10.18632/aging.102543 (PMC6949080; doi:10.18632/aging.102543)
Supplement: Supplementary Tables [file aging-11-102543-s003..pdf]

SUPPLEMENTARY TABLES

Please browse Full Text version to see the data of Supplementary Tables 1–3

Supplementary Table 1. Differential expression of miRNAs between malignant esophageal tissue and normal in GSE6188.

Supplementary Table 2. Differential expression of miRNAs between malignant esophageal tissue and normal in GSE43732.

Supplementary Table 3. Top pathways associated with 162 candidate targets of miR-203 in EC according to the enrichment analysis based on GO biological process.
